# Dose Optimization of Aditoprim-Sulfamethoxazole Combinations Against *Trueperella pyogenes* From Patients With Clinical Endometritis by Using Semi-mechanistic PK/PD Model

**DOI:** 10.3389/fphar.2021.753359

**Published:** 2021-11-16

**Authors:** Muhammad Kashif Maan, Tamoor Hamid Chaudhry, Adeel Sattar, Muhammad Abu Bakr Shabbir, Saeed Ahmed, Kun Mi, Waqas Ahmed, Shuyu Xie, Li Xin, Lingli Huang

**Affiliations:** ^1^ National Reference Laboratory of Veterinary Drug Residues/MAO Key Laboratory for the Detection of Veterinary Drug Residues, Wuhan, China; ^2^ Departement of Veterinary Surgery and Pet Sciences, University of Veterinary and Animal Sciences, Lahore, Pakistan; ^3^ Public Health Laboratory Division, National Institute of Health, Islamabad, Pakistan; ^4^ Department of Pharmacology and Toxicology, Faculty of Biosciences, University of Veterinary and Animal Sciences, Lahore, Pakistan; ^5^ Department of Microbiology, Faculty of Veterinary Medicine, University of Veterinary and Animal Sciences, Lahore, Pakistan; ^6^ Department of Biological Sciences, National University of Medical Sciences, Rawalpindi, Pakistan; ^7^ Department of Biomedical and Diagnostic Science, University of Tennessee, Knoxville, TN, United States; ^8^ MOA Huazhong Agricultural University, Wuhan, China

**Keywords:** PK/PD modeling, sulfamethoxazole resistance, aditorpim, combination therapy, Trueperella pyogenes, semi-mechanistic PK/PD modeling 3

## Abstract

Combinations of two and more drugs with different target sites are being used as a new treatment regimen for resistant clones of bacteria. Though, achieving the right combination of the drugs for optimal dosage regimen is challenging. In our study, we studied the antimicrobial effect of aditoprim, a novel dihydrofolate reductase inhibitor, and its synergistic effect with sulfamethoxazole. Synergy testing was performed by checkerboard micro dilution method and validation of different checkerboard ratios by static and dynamic time-kill analysis and *in vitro* pharmacokinetic/pharmacodynamics (PK/PD) model, and semi mechanistic PK/PD modeling was used to calculate and validate the synergistic effect of drug combination. Both checkerboard and static time-kill assays demonstrated the greater synergistic effect [fractional inhibitory concentration index (FICI) = 0.37] of the aditoprim [minimum inhibitory concentration (MIC) = 0.25 µg/ml]-sulfamethoxazole (MIC=>64 µg/ml) combination against all *T. Pyogenes* isolates. In the *in vitro* PK/PD model, the dosage proportion of sulfamethoxazole 4 mg/ml twice a day in combination with steady-state aditoprim 1 mg/ml efficiently repressed the growth of bacteria in 24 h with the ratio of 2-log10 decrease, related to the early inoculum against three *T. Pyogenes* isolates. The semi mechanistic PK/PD model projected that a combination of a high dose of aditoprim (2 mg/ml) with sulfamethoxazole (2 mg/day) was necessary to attain the killing of bacteria below the detection limit (limit of detection (LOD); i.e., 1 log10 CFU/ml) at 24 h with an MIC sulfamethoxazole (SMZ) of 64 µg/ml. However, it is anticipated that a combination of high dose of aditoprim with sulfamethoxazole is critical to attain the suppressed bacterial growth to < LOD. This study represents essential PK/PD modeling for optimization of combination of aditoprim and sulfamethoxazole to suppress growth of *T. Pyogenens*.

## 1. Introduction

Trueperella Pyogenes is a pathogen found in farm animals, recognized globally due to its commensal behavior that can cause subcutaneous abscesses, pneumonia, suppurative arthritis, and other associated infections in agriculture animals. A report from China has evidenced the progression of endocarditis in humans that live in close contact with animals, which proves it can also cause infections in human (Drillich, 2006). T. Pyogenes is a pathogenic environmental bacterium that typically causes an acute, purulent form of endometritis referred to as clinical endometritis. In animals, these pathogen usually locate on the skin surface, mucosa of the upper respiratory, and urogenital tracts (Rzewuska et al., 2019). Additionally, this bacterium was found in the microflora of bovine rumen, the gastrointestinal tract of swine (Jarosz et al., 2014), and also the microbiota of udder of healthy cattle (Galán-Relaño et al., 2020).

Antibiotics are precious resources which are essential in the fight against infectious agents (Stokes et al., 2020). Despite increases in the scientific research, there is a need for new antibacterial agents and to find out new antibiotic combinations to overcome the resistance problem in many pathogenic microorganisms of human and animals (Bollenbach, 2015; Tyers and Wright, 2019). T. Pyogenes is the major pathogenic bacteria causing gastrointestinal and urogenital infections in humans and animals (Bélanger et al., 2011; Pohl et al., 2018). Antimicrobial resistance against this organism is well documented in the literature (Tadesse et al., 2012). Despite many treatment options, considering the host environment, host drug interaction, and affectivity of the antibiotic agents, resistance against these microorganisms is still present and can cause treatment failure and devastating losses.

The increasing trend of multidrug resistance in bacterial clones all around the globe poses a severe threat to health systems. Therefore, combination antibiotic therapy is the best possible option to tackle serious infections. Presently, the synergistic effect of a combination is calculated by associating the detected and probable effects using a reference model. Keeping in view the resistance and choice of antimicrobial agents, the discovery of new antimicrobial agents is limited, which in turn limits the treatment option for clinical infections. However, in recent years the combination therapies against resistant bacteria have significantly increased to enhance the spectrum of activity of the antibiotics and increase the susceptibility of drug-resistant bacteria (Tyers and Wright, 2019). Indeed, experimental research to overcome antimicrobial resistance has provided strong evidence to program new antimicrobial drugs and new drug combinations to avoid the existence of resistant bacteria in the population (Ejim et al., 2011).

Aditoprim, as a selective folate reductase inhibitor, has been widely used in veterinary medicine as a potent antibacterial agent with wide spectrum against both gram positive and gram negative species. The antimicrobial spectrum of activity of aditoprim, like its congener trimethoprim, has been well documented in the literature (Cheng et al., 2017). Previously, trimethoprim in combination with sulfonamides such as sulfamethoxazole has been widely used in uterine infections due to the synergism between these two drugs (Guneysel et al., 2009). However, there is no evidence of the *in-vitro* antimicrobial susceptibility and synergistic action between aditoprim and sulfamethoxazole. Sulfonamides resistance among gram positive and gram negative bacteria is increasing globally (Sköld, 2000). The resistance patterns among these bacteria are also observed in other classes of antimicrobials. Treatment of multidrug-resistant bacteria has shown little clinical efficacy with aditoprim. The high morbidity and mortality due to the multidrug resistant *E. coli* has led to research for optimal antimicrobial combinations to maximize antimicrobial activity. *In-vitro* trimethoprim combination with sulfamethoxazole shows various degrees of synergy and increased bactericidal activity compared with trimethoprim alone (Nepka et al., 2016). As for resistance of emergence in trimethoprim combination therapy, there is a need for new antimicrobial agents like aditoprim in combination with sulfonamides to reduce and delay resistance development in time kill studies. *In-vitro* susceptibility data supporting these antibiotic combinations and optimal dosage regimens are lacking. There is no data supporting whether these antibiotic combinations act synergistically, additively, or antagonistically. Due to increasing trends of incidence of MDR pathogens all around the globe, it is suggested that we offer alternatives like mathematical models, metrics, pharmacodynamics (PD) modeling, and study strategies that can help in formulating effective and potent combinations with a concrete application in clinical setting (V.H. et al., 2015). During the modeling of antimicrobial combinations, the capacity to calculate interactions between these antimicrobials is a major task. The main terms used in these combinations and interactions are Synergism, additivity, and antagonism. We aimed to determine the aditoprim combination therapy with sulfamethoxazole for the treatment of *T. Pyogenes* infections in veterinary medicine. The purpose of this study was to determine the combination of aditoprim and sulfamethoxazole that revealed *in vitro* synergy by broth micro dilution checker board method. The combination of aditoprim and sulfamethoxazole were further studied by static time-kill assay and *in vitro* PK/PD model. The efficacy of aditoprim and sulfamethoxazole combination and their dosage regimen were evaluated by semi-mechanism-based PK/PD model.

## 2. Materials and Methods

### 2.1 Bacterial Strains, Experimental Design, and Susceptibility Testing

A total of 117 strains of pathogenic *T. Pyogenes* were isolated from infected cows and used for the study. Bacterial strains were identified on the basis of colony morphology of pathogenic *T. Pyogenes* on brain heart infusion (BHI) agar. Overnight grown cultures of isolates were subjected to molecular identification by targeting the specific gene (DSM 20630^T^) with specific universal oligonucleotide primers (5′-AGC​TTC​ACC​ACA​GCA​AGC​ACC​A-3′) by recommended conditions of PCR cycle as described previously (Hijazin et al., 2011). All the experimental isolates were kept in storage conditions of −80°C prior to DNA extraction.

According to guidelines provided by clinical laboratory standards in the institute (CLSI 2015), broth micro dilution (BMD) assay was employed for Antibiotic sensitivity testing of the isolates. An overnight incubation culture of bacterial strains was grown in MH (Mueller hinton) broth. After 6 h of incubation, 100 µl of the culture were placed in each well of the culture plate for MIC determination. Fresh isolates of *T. Pyogenes* were prepared in suspension, and concentration of bacterial suspension was adjusted according to 0.5 McFarland standards for determination of MIC. The end point where no bacterial growth was observed is considered as the MIC of the isolate and results were interpreted according to CLSI recommendations.

### 2.2 Synergy Testing by Broth Dilution Checkerboard Method

#### 2.2.1 Study Drugs and Preparation of the Inoculum

To carry out the experiment, aditoprim (ADP) (99% pure) and sulfamethoxazole (SMZ) (100 mg) was provided by the national reference laboratory for veterinary drug residues, HZAU, China and the national institute for food and drug control, China, respectively. For synergy testing by checkerboard (CB) method, an inoculum log10^6^ was grown in MH broth. After overnight incubation the turbidity was adjusted to 0.5 McFarland standards. 50 µl of the inoculum was used in each well for determination of the growth inhibition. Checkerboard is the combination of the two drugs in which aditoprim is serially diluted along the *x*-axis and SMZ was serially diluted along the *y*-axis. The starting concentration of both drugs was the MIC value of the certain drug against a particular bacterium. Different concentrations of antimicrobial drugs starting from the MIC value of each drug were used alone and in checkerboard combinations in customized 96-well micro titer plates against three standard strains of T. Pyogenes. Aditoprim was diluted in MH broth along the ordinate and SMZ was diluted in MH broth along the abscissa in 96 well micro titer plates. 100 µl volume of bacterial inoculum was used with the concentration of 1 × 10^6^ cfu/ml. Subsequently, plates were incubated overnight at 37°C.

A set of 20 isolates were selected from random sampling and subjected to anti-microbial sensitivity testing of ADP/SMZ combination at 1: 1, 1:2, 1:4, 1:8, and 1:16. To calculate the fractional inhibitory concentration (FIC), MIC of the drug combination was divided by the MIC of single drug. The sum of the FICs for ADP and SMZ in each combination was termed as the fractional inhibitory concentration index (FICI). If the FICI is more than one, it suggests the antagonism among both drugs. And if the FICI is less than one it indicates the synergism and the effect of the combination being greater than the sum of the effects of the individual drugs alone. The lower the FICI, the greater the synergism (Sopirala et al., 2010).

### 2.3 Static Time Kill Assay

Time-kill assay was executed for those ratios which showed synergy or additivity in checkerboard analysis. Using guidelines provided by National Committee for Clinical Laboratory Standards (NCCLS), T. Pyogenes isolates were incubated with 1 × 10^6^ cfu/ml concentration of the test strains (NCCLS C.L.S.I., 2007). Positive control was inoculated with isolate without any drug. After the allotted time intervals (i.e. 0, 3, 6, 12, 18, and 24 h), aliquots of 50 µl were removed and serial dilution of 1: 10 were performed with 450 ml of sterile saline. From each dilution, an inoculum of 20 µl was spread onto the surface of agar medium plates in the sequence of duplicates and cfu/ml was calculated by counting the viable colonies on agar plates after overnight incubation at 35°C (Hamoud et al., 2012). As evidenced by NCCLS, an antimicrobial agent which can achieve ≥3 × log10 (99.9%) reduction in colony forming units per ml (cfu/ml) after overnight incubation is bactericidal. And for the synergistic combination, the limit is a ≥2 × log10 reduction in growth.

### 2.4 Pharmacokinetic Analysis of Aditoprim-Sulfamethoxazole Combination

#### 2.4.1 Intra-uterine Administration of Aditoprim-Sulfamethoxazole

Cattle (*n* = 6) were infused with 50 ml of aditoprim-sulfamethoxazole at a fixed dosage regimen of 1 mg/kg of aditoprim, while sulfamethoxazole was given at a dosage regimen of 2 and 4 mg/kg with the help of an artificial insemination rod attached to a 10 ml syringe. The uterine fluid (1 ml) was collected in collecting tubes before and at 0.25, 0.5, 1, 3, 6, 9, 12 and 24 h post infusion in cattle. All uterine fluid samples were centrifuged at 5,000 ×*g* for 15 min at 4°C. All the samples were stored at −20°C until UV-HPLC analysis. All the sample tubes were properly marked with suitable identification numbers.

#### 2.4.2 Collection of Uterine Fluid

Caudal epidural anesthesia was performed with 2% xylocaine solution (10 ml; 100 mg) immediately before uterine fluid collection. Uterine horns were flushed non-surgically and flushing was always performed to the horn ipsilateral to ovary containing the CL first and flushing the contralateral horn afterward during post-estrus, A sterile silicone Foley catheter (2 vials, 20 ml cuff, 18″ or 20″ diameter; Rusch^®^, Teleflex, United States) was fixed onto a stylette. The catheter was passed through the cervix into the body of the uterus and directed to the target uterine horn by rectal palpation. The catheter was placed and secured at the horn’s bifurcation by inflating the cuff. The distal end of the uterine horn adjacent to the utero-tubal junction was manually blocked through rectal palpation. Then, the uterine horn was slowly filled with 30 ml of Dulbecco’s modified phosphate buffered saline (DMPBS, Nutricell, Campinas, United States) at 37°C using a sterile 50-ml catheter tip syringe. After infusion of DMPBS, the uterine fluid was immediately recovered by aspiration with a syringe. For visual aspect and recording the volume in each uterine horn, this procedure was repeated three times without removing the catheter.

### 2.5 *In vitro* PK/PD Model Experiments

For the execution of the kill-time experiment, the *in vitro* PK/PD model was used. Pharmacokinetics of drug combination was determined by high performance liquid chromatography (HPLC-UV) and the concentrations of both antimicrobials were simulated by the PK/PD model in the central compartment, which was well defined with a two-compartment model. Hence, the different doses of SMZ in the PK/PD model were simulated by mlxplore (version 2019Rb) with desire AUC/MIC ratio relevant to the time-concentration curve of SMZ in uterine fluid. Concentration of aditoprim was kept continuous at 0.5 or 1 mg/ml to simulate the steady-state concentration from the animals. *Ex vivo* dynamic time kill curves were obtained by exposure of the bacteria against different concentrations of SMZ obtained from the uterine fluid of cattle. Through syringe, samples were collected from the central compartment and, subsequently, bacterial count was performed by inoculation on Mueller-Hinton agar plates.

### 2.6 Semi-mechanistic PK/PD Modeling

As shown in the supplementary material, Supplementary Figure S3 represents a model scheme. k_g_ is the net growth rate of bacteria in a self-replicating state (B). ADP and SMZ in combination decrease the bacterial counts with the rate of k_drug_. C_ADP_ and C_SMZ_ symbolize the real-time concentration of aditoprim and sulfamethoxazole, respectively, while k_e_ represents the *in vivo* elimination rate of sulfamethoxazole.

#### 2.6.1 Bacterial Growth Modeling

Bacterial growth is a self-limiting process. A logistic function was used to describe the self-limiting process of bacterial growth which was characterized by the equation:

$$\frac{dB}{dt}=k_{g}\cdot \left(1-\frac{B}{B_{\max}}\right)\cdot B$$

Maximum bacterial count carry capacity is indicated by $B_{\max}$ in the system where B is the log colony counts of bacteria.

#### 2.6.2 Killing Effects of Aditoprim, Sulfamethoxazole, and the Combination

The change in the bacterial counts was described by the equation.

$$\frac{dB}{dt}=\left[k_{g}\cdot \left(1-\frac{B}{B_{\max}}\right)-E_{ADP}\right]\cdot B$$

Sigmoid E_max_ equation was used to describe the killing effects of the antimicrobial agent.

$$E_{ADP}=\frac{E_{\max\_ADP\cdot }C^{gADP}}{{\left(\alpha \cdot EC_{50\_ADP}\right)}^{gADP}+C_{ADP}^{gADP}}$$

$$\alpha =1+f.\left(1-e^{\left(-c_{ADP\cdot k\cdot t}\right)}\right)$$

E_ADP_ is the killing rate of aditoprim. E_max_ADP_ is the maximum achievable killing rate constant while EC_50_ADP_ is the antibiotic concentration that results in 50% of E_max_ADP_. An adaptation factor (*α*) is introduced in the equation which is dependent on time and drug concentrations to explain the adaptive resistance of aditoprim. The parameter of “*f”* and “*k”* is the maximal adaptation factor and rate of adaptation, respectively. The increase of α could result in an increase in EC_50_ADP_.

The killing effect of sulfamethoxazole against *T. Pyogenes* was consistent with the following sigmoid E_max_ model

$$\frac{dB}{dt}=\left[k_{g}\cdot \left(1-\frac{B}{B_{\max}}\right)-E_{SMZ}\right]\cdot B$$

$$E_{SMZ}=\frac{E_{\max\_SMZ}\cdot C_{SMZ}^{gSMZ}}{{\left(EC_{50\_SMZ}\right)}^{gSMZ}+C_{SMZ}^{gSMZ}}$$

where $EC^{50\_SMZ}C^{50\_SMZ}$ is the antibiotic concentration that results in 50% of. $E_{\max\_SMZ.}$

The drug interaction in the combination therapy was evaluated using the following equation

$$E={\left(\frac{E_{\max\_ADP}\cdot C^{gADP}}{EC_{50\_ADP}+C_{ADP}^{g}}\right)}^{\mathrm{int}}+{\left(\frac{E_{\max\_SMZ}\cdot C^{gSMZ}}{EC_{50\_SMZ}+C_{SMZ}^{g}}\right)}^{\mathrm{int}}$$

where E is the killing rate constant of aditoprim and sulfamethoxazole in combination. Int represents the interaction effect which indicates the effect of synergy, indifference, and antagonism. A positive value indicates a synergistic effect, a negative value indicates an indifference or antagonism effect, and zero indicates no interaction (Bian et al., 2019).

#### 2.6.3 Analysis of Data

Model fittings were performed by nonlinear regression analysis using the maximum likelihood algorithm in Matlab (R2018b) (Mathwork, Inc., United States). Parameter estimates were obtained after model fitting. To investigate the effect of different dosage regimens, the pharmacodynamics model describing the bacterial growth rate in the function of sulfamethoxazole concentration was combined with the PK model and simulation were performed with mlxplore software (version 2019R1, Lixoft, Orsay, France). Data obtained from the above experiment were analyzed by prism graph pad software (verion7.0). Mean and standard deviation were calculated by test variables. Comparison of different ratios of both drugs for determination of synergy was determined by analysis of variance (ANOVA). *p* = <0.05 is considered as significant.

## 3. Results

### 3.1 Susceptibilities, Checkerboard, and Time-Kill Studies

The present study was conducted to evaluate the antimicrobial effect of aditoprim and sulfamethoxazole and their synergistic action against T. pyogenes. The minimum inhibitory concentration (MIC) of aditoprim (ADP) and sulfamethoxazole (SMZ) ranged between 0.25 and 8 µg/ml and 64 to 128 µg/ml, respectively. Supplementary Figure S1 of supplementary material showed the MIC distribution of T. Pyogenes isolates for ADP and SMZ. According to MIC values, T. pyogenes was sensitive to ADP (MIC = 0.25 µg/ml) but it was resistant to SMZ (MIC = 128 µg/ml).

In the checkerboard analysis, three reference strains of T. pyogenes were used to check the synergy between ADP and SMZ. The MIC values and the results of different checkerboard combinations of two drugs were shown in Table 1. The FICI of two drug combinations showed the additive interaction between two drugs. But at the ¼ MIC of ADP and ¼ MIC of SMZ the two drugs showed synergism. This showed the activity of the ADP in combination with SMZ below its therapeutic range.

**TABLE 1:** FICI values of ADP and SMZ checkerboard combination against three standard strains of *T. Pyogenes*.

Strain	MIC ADP (µg/ml)	MIC SMZ (µg/ml)	FICI	Interpretation
Strain 1	8	0	0	—
	4	4	0.62	Additivity
	2	8	0.37	Synergy
	1	16	0.62	Additivity
	0.5	32	1.06	Indifference
	0.25	64	1.03	Indifference
	0	128	0	—
Strain 2	8	0	0	—
	4	8	0.62	Additivity
	2	16	0.37	Synergy
	1	32	0.62	Additivity
	0.5	64	1.06	Indifference
	0.25	128	1.03	Indifference
	0	256	0	—
Strain 3	8	0	0	—
	4	8	0.62	Additivity
	2	16	0.37	Synergy
	1	32	0.62	Additivity
	0.5	64	1.06	Indifference
	0.25	128	1.03	Indifference
	0	256	0	—

Supplementary Table S1 of supplementary material showed the FIC index value for different ratios of ADP and SMZ (1:1, 1:2, 1:4, 1:8 and 1:16) against 20 randomly selected isolates of T. pyogenes. There was a significant synergy and additivity (*p* = 0.0007) between two drugs in different combinations for randomly selected T. Pyogenes isolates. According to the analysis of variance (ANOVA), there was no significant difference between 1:1 and 1:2 (*p* = 0.8) as these two ratios inhibit the growth of only 2 and 5 isolates from 20 randomly selected isolates. Similarly, there was no significant difference between ratio 1:1 and 1:4 (*p* = 0.2), 1:2 and 1:4 (*p* = 0.96), and 1:8 and 1:16 (*p* = 0.3). There was significant difference when ratio 1:8 and 1: 16 were compared with other ratios as these two ratios inhibited the growth of 13 and 17 from randomly selected T. Pyogenes isolates respectively (Supplementary Table S2). From these results it was suggested that ratio 1:8 and 1:16 were more synergistic (*p* = 0.0001) compared to the other ratios of ADP and SMZ.

**FIGURE 1:**
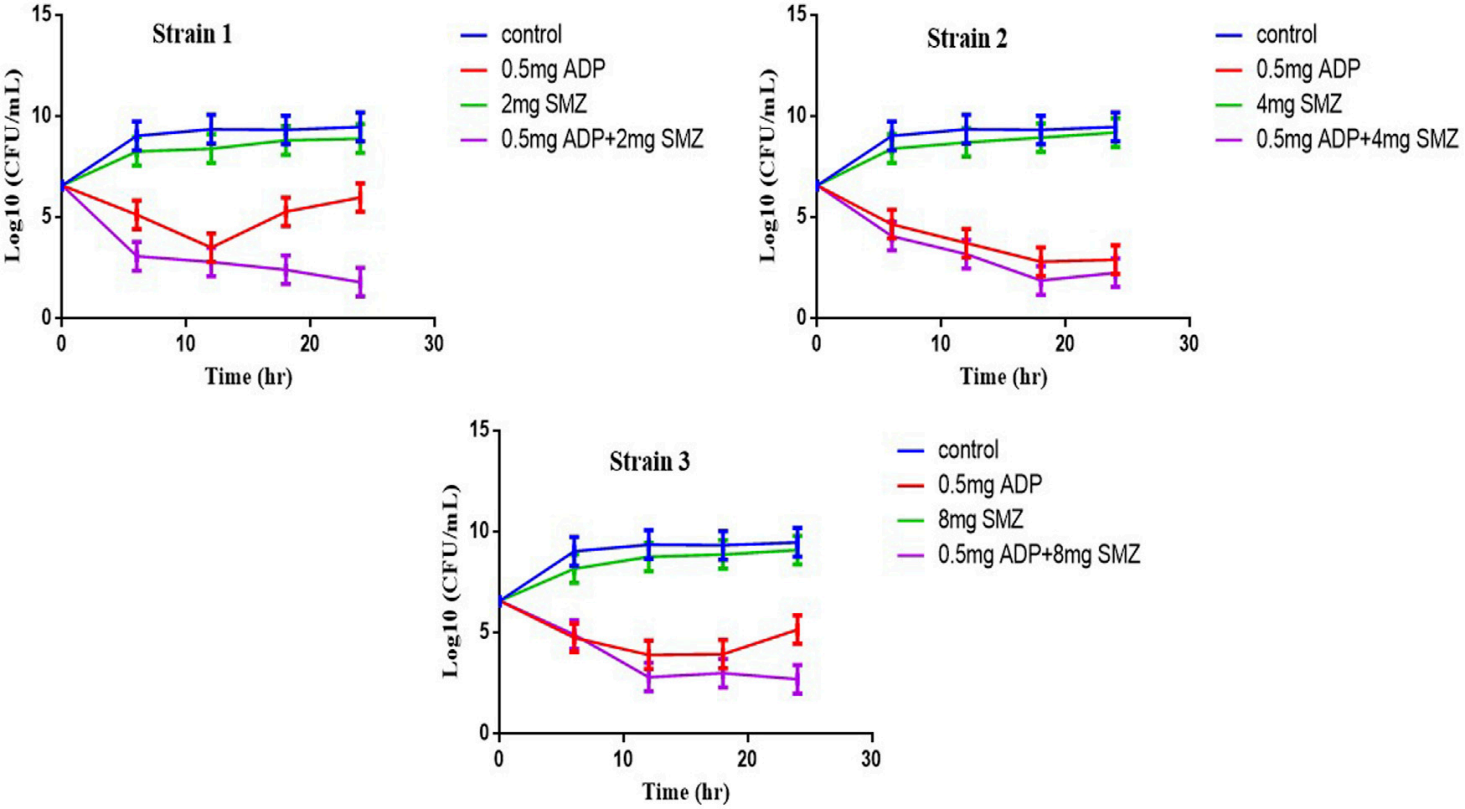
Static time-kill curves show the bactericidal effect of aditoprim (red), sulfamethoxazole (blue) and their combination (purple) against *T. Pyogenes* (mean ± SD, *n* = 3) by broth micro dilution method according to the NCCLC guidelines. ADP, aditorim; SMZ, sulfamethoxazole.

Three strains of T. pyogenes were selected for the static time-kill analysis. Lowest fractional inhibitory concentration indexes (FICIs) were used to choose the drug concentration in time-kill study. The time-kill curves and the logCFU_0 –24_ of each regimen were shown in Figure 1 and Table 2. Sulfamethoxazole alone did not show a killing effect on bacteria due to the high MIC value. However, after combining with ½ or ¼ MIC of aditoprim, there was pronounced killing effect at 3–6 h and regrowth was observed afterwards with the same logCFU_0–24_ in control group at 24 h. Strong synergistic effect was observed in combination therapy with logCFU_0–24_ being -5.33, -2.3, and -5.28 for three strains of *T. Pyogenes.* Time-kill study was also used to validate the synergy between the significantly synergistic ratios of ADP and SMZ by checkerboard method. 1:8 and 1:16 showed synergy at 6 and 24 h by decrease in the bacterial count when compared to the ADP monotherapy.

**TABLE 2:** logCFU_0–24_ values of aditoprim and sulfamethoxazole as monotherapy and in combination.

Bacterial strains	MIC(ADP/SMZ)	logCFU_0–24_ by antibiotic therapy (mean ± SD) (*n* = 3)			
		No drug	Aditorpim	Sulfamethoxazole	Combination
Strain 1	(0.5/64)	3.39±0.17	0.02±0.005	0.64±0.05	−5.33±0.31
Strain 2	(0.5/128)	2.91±0.11	1.22±0.06	1.59±0.11	−2.3±0.2
Strain 3	(0.5/128)	3.32±0.19	0.62±0.03	1.93±0.20	−5.28±0.15

### 3.2 *In vitro* PK/PD Model

Pharmacokinetics of both antimicrobial agents was determined by the PK model into the central compartment. Sulfamethoxazole concentration was simulated at the dosage regimen of 2 and 4 mg/kg in healthy cows and PK parameters were provided in Supplementary Table S3 in the supplemental material. The uterine fluid concentration-time profile is shown in Supplementary Figure S2 of the supplementary material. After intra uterine administration of ADP-SMZ compound injection, uterine fluid concentration was described by non-compartmental pharmacokinetic model.

**FIGURE 2:**
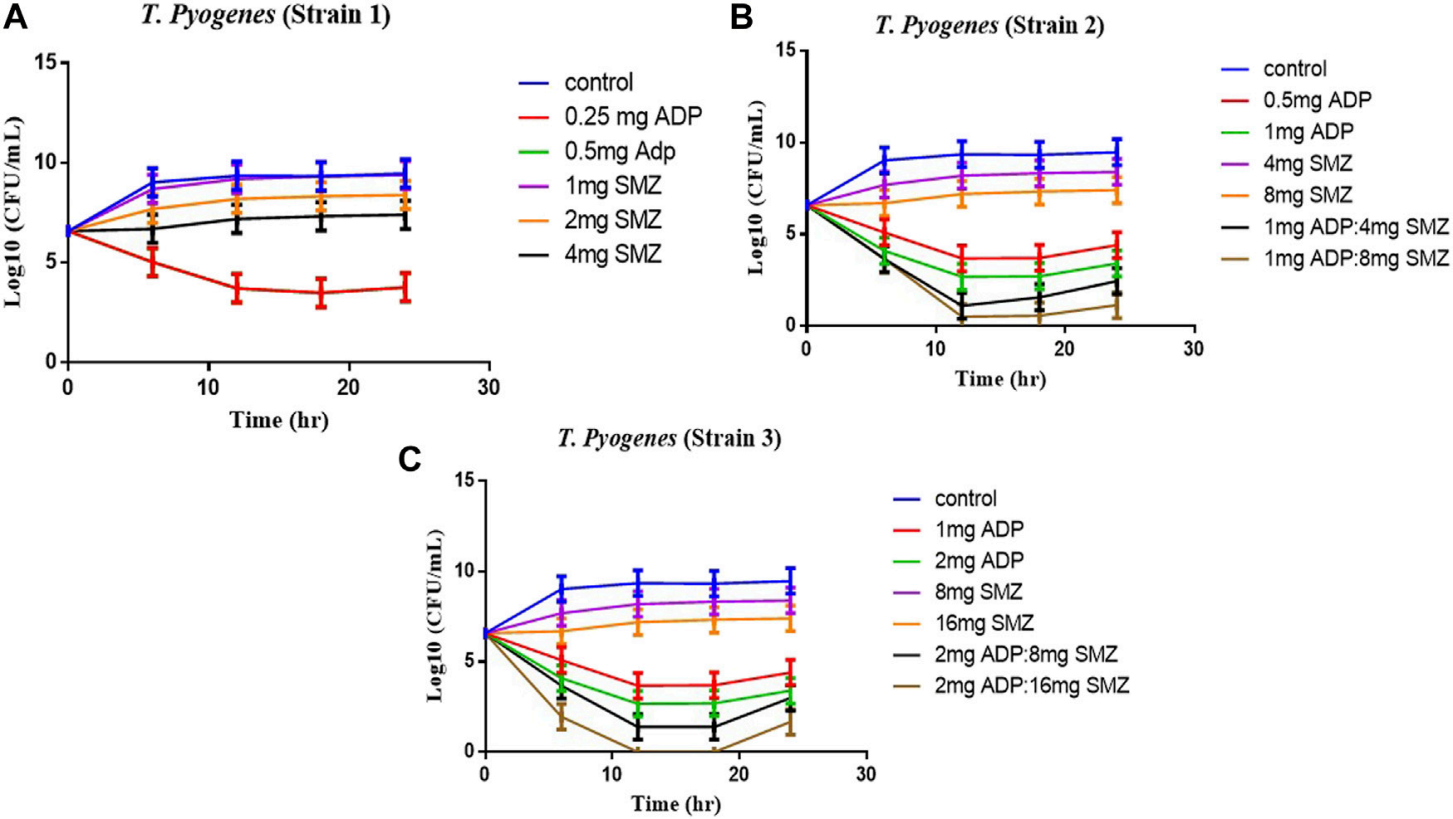
Dynamic *in vitro* killing kinetics of Strain1 **(A)**, Strain2 **(B)** and Strain3 **(C)** in different dosage regimens of aditoprim and sulfamethoxazole mono and in combination therapy. The decrease in logCFU_0–24_ of *T. Pyogenes* (mean ± SD, *n* = 3) with time for mono and combination therapy of ADP and SMZ demonstrate synergy between two drugs.

**FIGURE 3:**
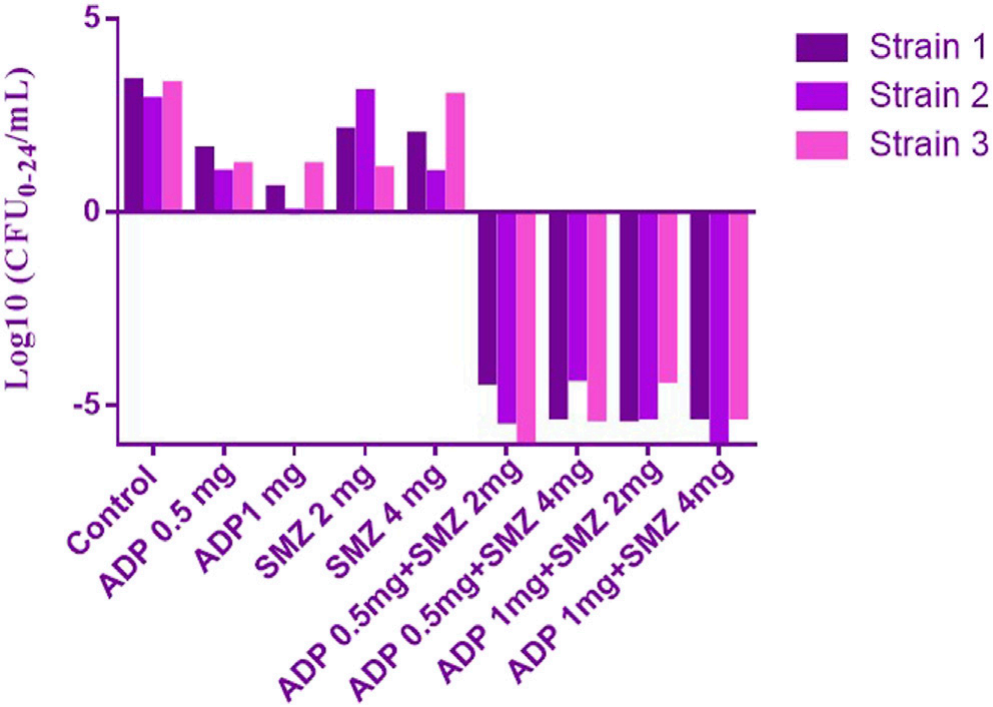
The values of logCFU_0–24_ for three strains of *T. Pyogenens* after each mono and combination therapy. The value of logCFU_0–24_ demonstrates the killing effect of aditoprim and sulfamethoxazole mono and combination therapy against *T. Pyogenens* (*n* = 3).

Time-kill curves under different regimens of aditoprim and sulfamethoxazole alone or in combination were shown in Figure 2. The logCFU_0–24_ values were shown in Figure 3. At the concentration of 0.25, 0.5, and 1 mg/ml, aditoprim showed rapid bacterial killing within 6–12 h (Figure 2) with logCFU_0–t_ of -1.05, -2.03, and -3.7 (Figure 3). However, there was regrowth after 12 h and no difference was found at 24 h between control group and aditoprim monotherapy. In sulfamethoxazole monotherapy, there were different bactericidal effects according to the MIC of the bacterial strains. For bacterial stain (MIC = 64 µg/ml), 4 mg SMZ killed bacteria and no bacterial colony was detected at 24 h. For Strain 3 (MIC_SMZ, 128 µg/ml), there was no decrease in bacterial count during 24 h. The AUC/MIC values at different time intervals of concentration-time curve did not meet the desired MIC breakpoint of low and high MIC bacterial strains after 2 and 4 mg/ml of sulfamethoxazole administration (Table 3 and Table 4).

**TABLE 3:** Values of AUC/MIC corresponding to concentration-time curve for *T. Pyogenes* at 2 mg/kg dose of sulfamethoxazole.

Time	SMZ (2 mg)	AUC	AUC/MIC (64)	AUC/MIC (128)
0.5	82.88	0	0	0
1	69.6	38.12	0.595,625	0.297,813
2	50.56	98.2	1.534,375	0.767,188
4	33.6	182.36	2.849,375	1.424,688
6	25.44	214.4	3.35	1.675
8	19.36	286.2	4.471,875	2.235,938
12	14.4	353.72	5.526,875	2.763,438
24	4.8	468.92	7.326,875	3.663,438

**TABLE 4:** Values of AUC/MIC corresponding to concentration-time curve for *T. Pyogenes* at 4 mg/kg dose of sulfamethoxazole.

Time	SMZ (4 mg)	AUC	AUC/MIC_64	AUC/MIC_128
0.5	331.52	0	0	0
1	278.4	152.48	2.3825	1.19125
2	202.24	392.8	6.1375	3.06875
4	134.4	729.44	11.3975	5.69875
6	101.76	965.6	15.0875	7.54375
8	77.44	1,144.8	17.8875	8.94375
12	57.6	1,414.88	22.1075	11.05375
24	19.2	1875.68	29.3075	14.65375

For bacterial strains (MIC_SMZ, 128 µg/ml), there was no bactericidal effect in sulfamethoxazole monotherapy. Combination therapy revealed a stronger killing effect against *T. Pyogenes* than monotherapy. For strain 1, 1 mg/ml aditoprim in combination with I mg/ml sulfamethoxazole produced 4-log10 killing during 24 h. However, regrowth occurred at 24 h. When SMZ was increased to 2 mg in the combination, there was no sustainable colony at 6 h, and logCFU_0–24_ was -5.38 at 24 h. For strain 2 and 3, the combination therapy produced a strong bactericidal effect in the first 6 h and the logCFU_0–24_ was 2.13 and -1.69, respectively, when 1 g and 2 mg SMZ was used in combination.

### 3.3 Semi-mechanistic PK/PD Model

**FIGURE 4:**
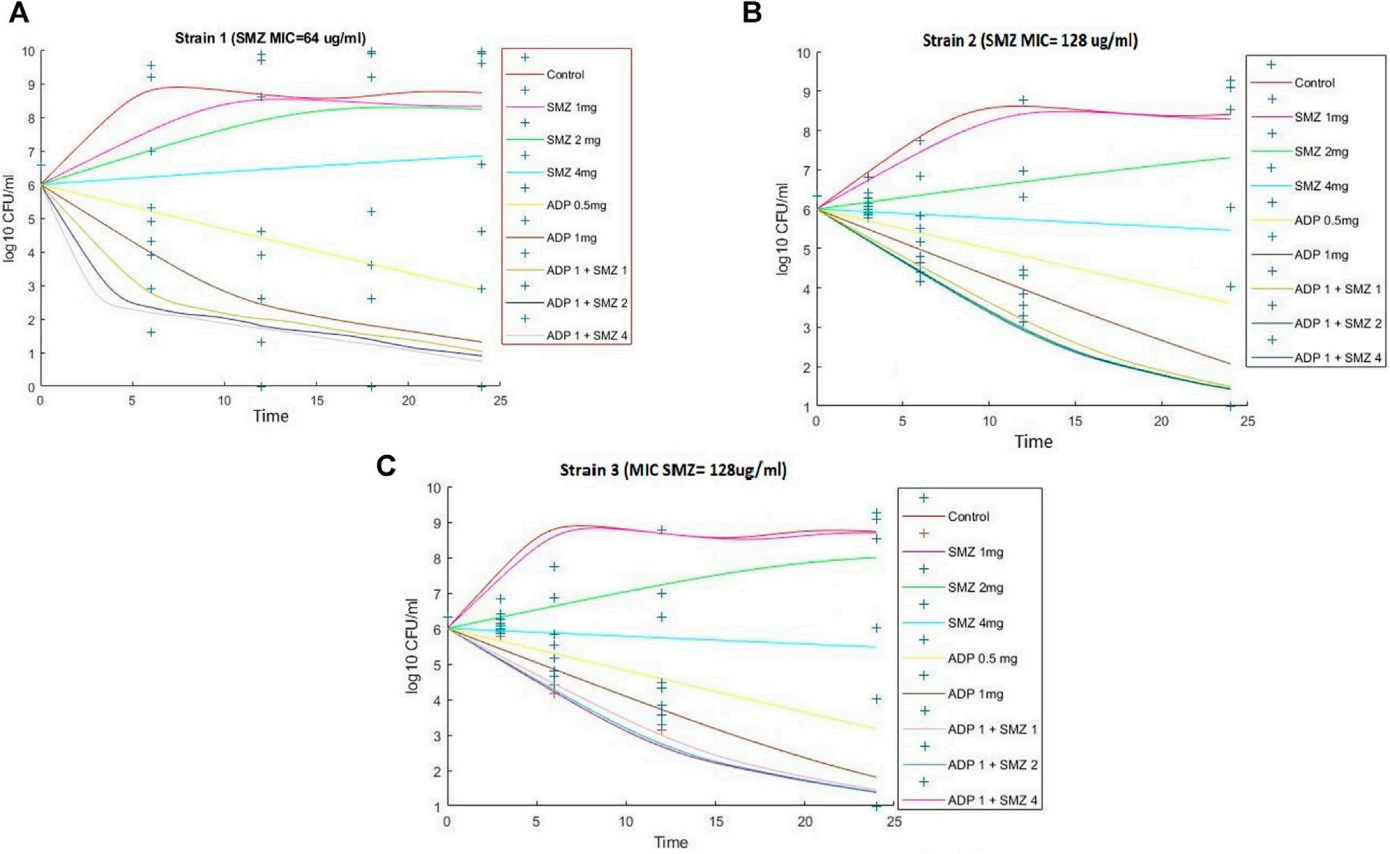
Observed (symbols) and model fitted (lines) viable counts for the dynamic *in vitro* PK/PD model experiments with aditoprim or sulfamethoxazole alone and the combination against *T. Pyogenes* (Strain 1) **(A)**, Strain 2 **(B)**, and Strain 3 **(C)**. Sulfamthoxazole dose of 2 or 4 mg is infused for 3-h infusion. ADP, aditoprim; SMZ, sulfamethoxazole.

**FIGURE 5:**
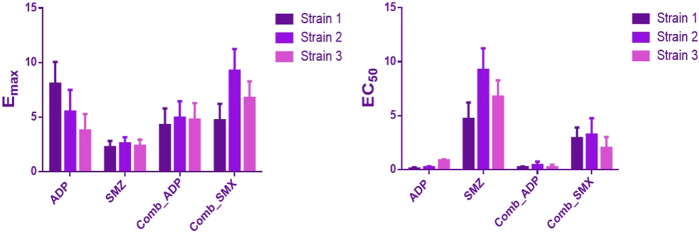
Comparison of estimated parameters E_max_ and EC_50_ for the three strains of *T. Pyogenes* (mean ± SD, *n* = 3) in the static time-kill study. ADP and SMZ represent aditoprim and sulfamethoxazole parameters in monotherapy; COMB_ADP and COMB_SMZ represent aditoprim and sulfamethoxazole parameters in the combination therapy (*p* < 0.05).

**FIGURE 6:**
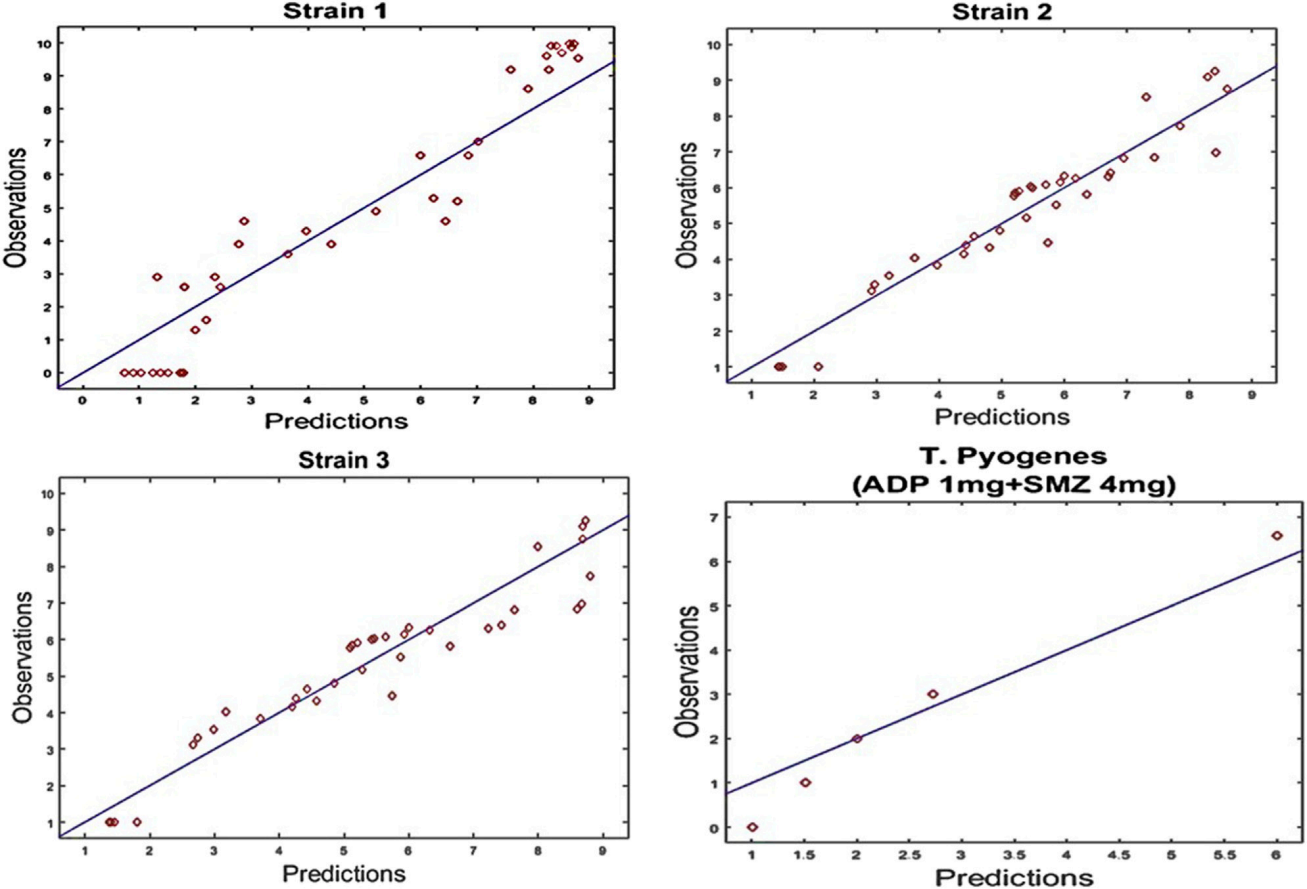
Diagnostic plots of fitting results of time kill curves data for strain1, Strain2 and strain3 of T. Pyogenes and validation of results for strain1 at dose regimen of 1 mg/ml aditoprim in combination with 4 mg/ml of sulfamethoxazole. Diagnostic plots indicated that results were well fitted and symmetrical distribution of the data point closed to the regression line.

In semi-mechanistic PK/PD model, static time-kill curve data were applied. Both experimental data and modeling simulation curves were adequately described by the proposed PK/PD model. In the dynamic *in vitro* PK/PD model, all experimental data of time kill curves were fitted for parameter estimation (Figure 4). Typical parameter estimates were presented in Table 5. Aditoprim did not show significant difference in the values of EC_50_ and E_max_ in mono and combination therapy. However, sulfamethoxazole in combination showed lower EC_50_ value than monotherapy, which was 1.92 mg/ml versus 7.24 mg/ml (Figure 5). Diagnostic plots of predicted versus actual values were obtained which indicated that results were well fitted, and symmetrical distribution of the data point were close to the regression line (Figure 6).

**TABLE 5:** Parameter estimates for the *in vitro* PK/PD model.

Parameters	Units	Strain 1	Strain 2	Strain 3
B_max_	log10 CFU/ml	9.60E+00	8.46E+00	8.00E+00
k_growth_	1/h	1.46	2.01	1.64
E_max_ADP_	1/h	8.06	3.49	2.09
EC_50_ADP_	mg/ml	0.017	0.11	0.95
g__ADP_	—	0.49	1.02	0.98
E_max_SMZ_	1/h	1.9	2.92	2.02
EC_50_SMZ_	mg/ml	0.12	7.51	0.01
g__SMZ_	—	11.14	2.05	1.96
Int	—	0.71	0.65	0.17
*f*	—	1.9	2.5	8.45
*k*	—	1.02	0.25	0.54

### 3.4 Model Validation and Prediction

**FIGURE 7:**
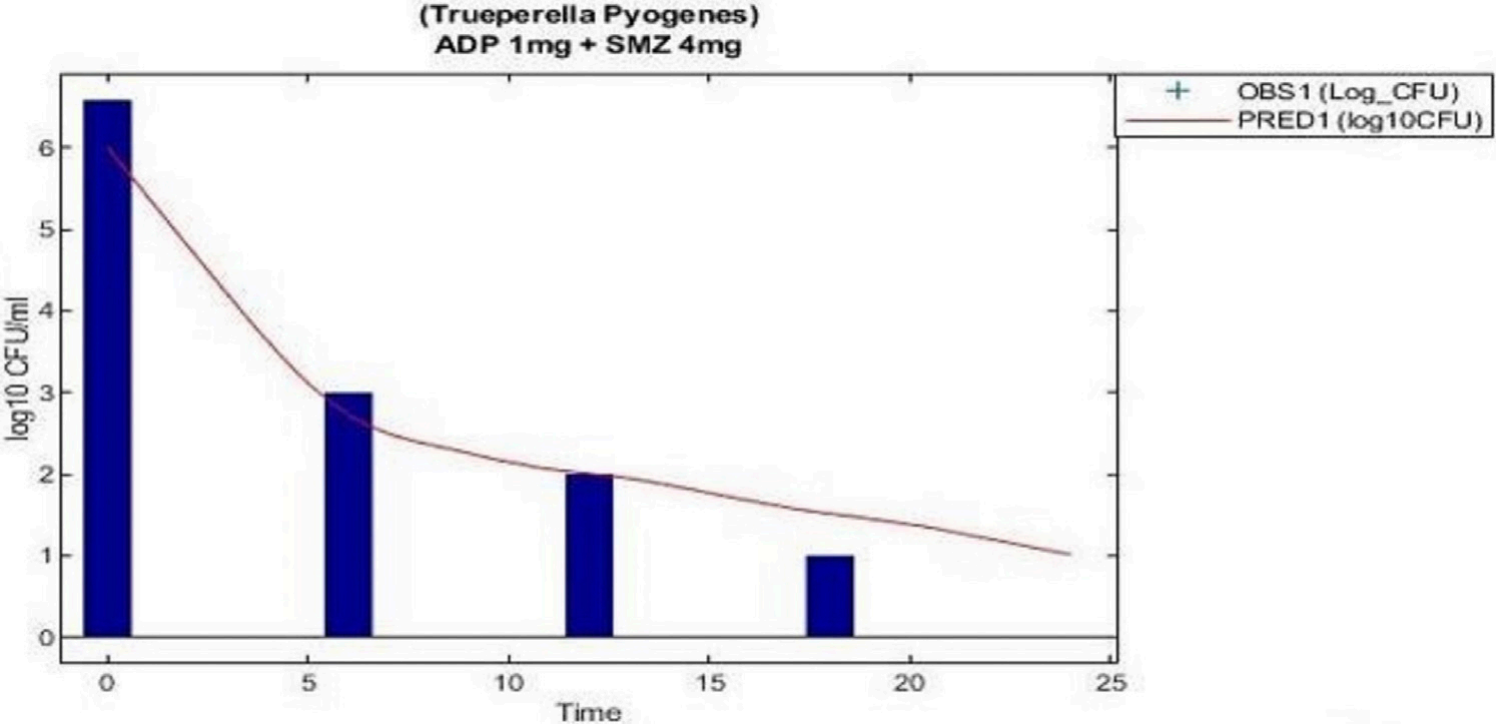
Validation of the PK/PD modeling for the regimen of 1 mg/ml aditoprim in combination with 4 mg/ml with 3 h infusion of sulfamethoxazole.

**FIGURE 8:**
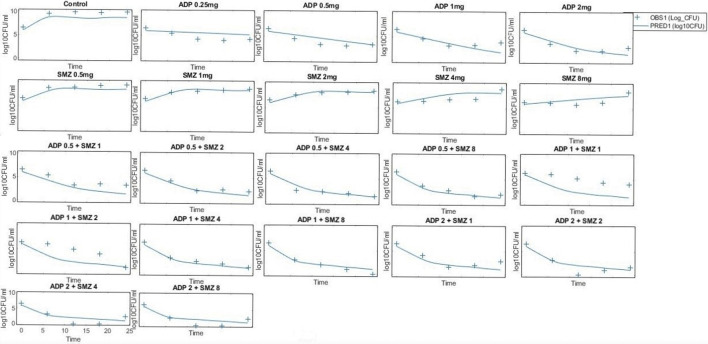
Pharmacodynamic predictions of aditoprim and sulfamethoxazole mono- and combination therapy against *T. Pyogenes*. During monotherapy, aditoprim (2 mg/ml) killed most of the bacteria while efficacy of sulfamethoxazole did not show pronounced effect with increased in dose. However, in combination of two drugs, there was an increase in bactericidal effects for all dosing combinations. The units for aditoprim and sulfamethoxazole are mg/ml at steady state with 3-h infusion. LOD, 1 log10 CFU/ml.

The model predicted results of aditoprim (1 mg/ml) and sulfamethoxazole (4 mg/ml) in combination therapy was simulated and were shown *via* a solid line close to observed values. However, after 12 h regrowth occurred, which was lower than experimental data (Figure 7). Parameters from the dynamic experiments were used for the predictions of bactericidal effects of new therapies (Figure 8). During monotherapy, aditoprim (2 mg/ml) killed most of the bacteria while efficacy of sulfamethoxazole did not show a pronounced effect with increases in dose. However, in combination of two drugs, there was an increase in bactericidal effects for all dosing combinations. 2 mg/ml aditoprim in combination with 8 mg/ml sulfamethoxazole, which were highest in concentration, showed bacterial counts close to the limit of detection (LOD) at 24 h. For strain 1, aditoprim (2 mg/ml) showed a stronger bactericidal effect than sulfamethoxazole which can barely kill bacteria even with the highest dosage.

## 4. Discussion

In the present study, a combination of aditoprim and sulfamethoxazole was developed in a semi-mechanistic PK/PD model and both static and dynamic time-kill studies were conducted for *T. Pyogenes*. The model of two antimicrobial combinations is well described and the time course of bacterial growth reduction was predicted, which is applicable as a significant combination therapeutic regime in healthcare settings.

The objective of this *in vitro* study was to evaluate the synergy between ADP and SMZ that are commonly used clinically for the treatment of infectious disease in veterinary medicine by checkerboard method and validation of the results by time-kill analysis. In our study, aditoprim, a novel dihydrofolate reductase, in combination with sulfamethoxazole, has a synergistic effect with trimethoprim (which is also a dihydrofolate reductase inhibitor similar to aditoprim). We validate the synergy between ADP and SMZ by time-kill analysis against clinical isolates of pathogenic *T. Pyogenes* (Figure 1 and Figure 2). Bacterial isolates were tested for sensitivity determination for ADP and SMZ. *T. Pyogenes* was sensitive for ADP (MIC = 0.25 µg/ml) but these MIC values were slightly higher than that found by Cheng et al., 2017. In our study, *T. Pyogenes* isolates were more susceptible to both drug combinations than single drug. Both drugs showed synergy by checkerboard (CB). In checkerboard (CB) analysis the MIC of both drugs reduced to ½ to ¼ by the original MIC value which suggest the strong synergism between two drugs. These results were in accordance with another study (Vilchèze and Jacobs, 2012; Nepka et al., 2016) which evaluated the effectiveness of trimethoprim:sulfamethoxazole (TMP:SMX) combination against *tuberculosis*. Both drugs found, as did we, that at the ratio between 1:1, 1:2, 1:4, 1:8, and 1:16 major reduction effects were had on the *in vitro* action against *T. Pyogenes* isolates. V.H. et al., 2015 proposed the efficient ratio of a two drugs combination should relate to the maximal effective ratio of a single drug alone, showing growth reduction of bacterial isolates. Approximately 95% of the isolates in the study had the MIC ratio of ADP and SMZ combination ranging from 1:1 to 1:64, with the majority between 1:8 and 1:16. Therefore we might expect the optimum ratio would be between1:4 to 1:8. With time-kill testing, decreases in viable count were observed at 6 and 24 h for 1:8 and 1:16 ratio of ADP and SMZ. Time-kill study validates the synergy by checkerboard (CB) method. SMZ is a concentration dependent antibacterial agent. AUC/MIC is a PK/PD parameter to determine the efficacy of concentration dependent antimicrobial agents. In our study, AUC/MIC ratio at different time points of concentration-time curve did not achieve the desired clinical breakpoint due to the high MIC values of the three strains of *T. Pyogenes* (Table 3, Table 4)*.* These results suggested the higher concentrations of the SMZ required to achieve desired pharmacodynamics goals. However, when different doses of SMZ were combined with a fixed dose of ADP (0.5 or 1 mg/ml), the killing effect was enhanced. Similar findings have been observed in other studies (Autmizguine et al., 2018), in which the exposure achieved was matched in children and adult groups where TMP-SMX was orally administered after each 12 h interval at a dose rate of 8/40 and 320/1600 mg/kg of body weight/day respectively. The PD target for bacteria was achieved with an MIC of 0.5 mg/L in >90% of infants and children. In the PK/PD model, the achieved value of growth rate for strain 1 was 1.46/h and 2.01/h for strain 2. It has been recognized that an induced resistant strain decreases the growth rate when linked with that of their susceptible parental strains. In the prediction section, it was revealed that higher growth of bacteria was more challenging to kill to the limit of detection level. Hence, to kill bacteria with higher growth rate, a bactericidal with a higher dose was required rather than higher MICs.

Similarly, aditoprim belongs to a concentration-dependent antimicrobial agent. Due to the low volume of distribution, it is not widely distributed in body fluids and tissue and has a long half -life as previously reported in pigs (Wang et al., 2016). However, it had been observed in our study that the *ex-vivo* antibacterial pattern of aditoprim had some alteration and was different from the pattern of *in-vitro* conditions in time-killing curve experiment (Figures 2, 3). The activity of bactericidal is more efficient and fast if it is concentration dependent instead of time dependent (Czock et al., 2009), as the concentration in uterine fluid increased, the bacterial counts had dropped after 24 h incubation, indicating a concentration-related antibacterial activity under *ex-vivo* condition. Similar findings had been reported by Yan et al., 2017, as a concentration-related killing pattern of bacterial counts decrease in ilium contents was revealed by oral administration of cyadox in pigs. For the strains with ADP MIC ≤ 1 µg/ml, it indicated that level of ADP in uterine fluid sustained at 1 mg/ml combined with 4 mg of sulfamethexazole with 3 h duration could be able to reduce initial inoculum with the rate of _2- to 3-log10 reduction.

Semi-mechanistic PK/PD model was employed on the basis of the antibacterial properties of aditoprim and sulfamethoxazole against *T. pyogenes*. The simulation profiles of aditoprim manifested an evident association among the observed and estimated profile with the *ex-vivo* antibacterial efficacy. In the semi-mechanistic model, the E_max_ of the aditoprim did not fluctuate in mono and combination therapy. But in the case of sulfamethoxazole, the EC_50_ values dropped in combination therapy as compared to monotherapy, suggesting the enhanced killing rate of bacteria when combined with the aditoprim. These findings were in accordance with the previous study, in which sulfamethoxazole in combination with trimethoprim enhanced the killing rate of bacteria.

To sum up, this study establishes the potential of two drug combinations against *T. pyogenes*. The strong synergism between these drugs suggested their therapeutic potential. All the combinations showed synergy in checkerboard (CB) and time-kill assay. On the other hand, most of these combinations showed additivity in checkerboard (CB) was also easier to perform, less time consuming, and less expensive. The clinical benefits of these antibiotic combinations *in vivo* can only be determined by assessing synergies through carefully designed pharmacokinetic studies and randomized clinical trials. Efficacy of different dosage regimens in combination can be predicted by *in vivo* experiments.

## Data Availability

The original contributions presented in the study are included in the article/Supplementary Material, further inquiries can be directed to the corresponding author.
