# Supplementary material for: Dose Optimization of Aditoprim-Sulfamethoxazole Combinations Against Trueperella pyogenes From Patients With Clinical Endometritis by Using Semi-mechanistic PK/PD Model
Source: Front Pharmacol. 2021 Nov 16;12:753359. doi: 10.3389/fphar.2021.753359 (PMC8635024; doi:10.3389/fphar.2021.753359)
Supplement: Supplementary file 1 [file DataSheet1.docx]

**Dose optimization of aditoprim-sulfamethoxazole combinations against *Trueperella pyogenes* from patients with clinical endometritis by using semi-mechanistic PK/PD model**

Muhammad Kashif Maan^1,7^, Adeel Sattar^4^_,_ Muhammad Abu Bakr Shabbir^3^, Saeed Ahmed^6^, Kun mai^1^, Waqas Ahmed^5^_,_ Shuyu Xie^1^, Li Xin^1^, Lingli huang^1,,2**^

^1^National Reference Laboratory of Veterinary Drug Residues/ MAO Key Laboratory for the Detection of Veterinary Drug Residues, ^2^MOA Huazhong Agricultural University, Wuhan 430070, PR China

^3^Department of Microbiology, Faculty of Veterinary Medicine, University of Veterinary and Animal Sciences, Lahore, Pakistan

^4^Department of Pharmacology and toxicology, Faculty of Biosciences, University of Veterinary and Animal Sciences, Lahore, Pakistan

^5^Department of biological sciences, Old Dominion university, Norfolk, USA

^6^National university of medical sciences, Pakistan

^7^Departement of Veterinary Surgery and Pet Sciences, University of Veterinary and Animal Sciences, Lahore, Pakistan

**Running title:** Semi-mechanistic PK/PD modeling and dose optimization

**Correspondence**: Lingli Huang

(huanglingli@mail.hzau.edu.cn)

**Table S1** FIC index for 20 isolates of *T. Pyogenes*. Each value represents mean ± standard deviation

| **Ratio**  **ADP/SMZ** | **FIC index** |
| --- | --- |
| 1:1 | 0.6±0.04 |
| 1:2 | 0.56±0.120 |
| 1:4 | 0.58±0.068 |
| 1:8 | 0.4±0.044 |
| 1:16 | 0.35±0.090 |

**Table S2** Comparison of different ratios of ADP and SMZ by ANOVA. P<0.5 consider as significant

| Ratios | Significant | | P value |
| --- | --- | --- | --- |
| 1:1 vs. 1:2 | No | ns | 0.8167 |
| 1:1 vs. 1:4 | No | ns | 0.2069 |
| 1:1 vs. 1:8 | Yes | **** | <0.0001 |
| 1:1 vs. 1:16 | Yes | **** | <0.0001 |
| 1:2 vs. 1:4 | No | ns | 0.9608 |
| 1:2 vs. 1:8 | Yes | **** | <0.0001 |
| 1:2 vs. 1:16 | Yes | **** | <0.0001 |
| 1:4 vs. 1:8 | Yes | **** | <0.0001 |
| 1:4 vs. 1:16 | Yes | **** | <0.0001 |
| 1:8 vs. 1:16 | No | ns | 0.3636 |

**Table S3** Pharmacokinetic parameters of sulfamethoxazole

|  |  |  |  | **Dose** |  |
| --- | --- | --- | --- | --- | --- |
| **Parameters** | |  | **Unit** | **2 mg** | **4mg** |
| **Two compartmental analysis** | | |  |  |  |
| V |  |  | (L) | 0.024 | 0.0121 |
| k_12_ |  |  | (h-1) | 0.211 | 0.211 |
| k_21_ |  |  | (h-1) | 0.335 | 0.335 |
| Ke |  |  | (h-1) | 0.08 | 0.08 |
| K |  |  | (h-1) | 0.164 | 0.164 |
| **Non compartmental analysis** | | |  |  |  |
| AUC_0-24_ |  |  | ug/h/ml | 468.92 | 1875.68 |
| C_max_ |  |  | ug/ml | 82.88 | 331.52 |
| T_max_ |  |  | H | 0.5 | 0.5 |
| T_1/2_ |  |  | H | 7.86 | 7.86 |
| MRT |  |  | H | 6.59 | 6.59 |
| V_ss_ |  |  | L/kg | 0.043 | 0.021 |
| CL |  |  | 1/kg/h | 0.003 | 0.0019 |

**Figure S1** MIC distribution of aditoprim and sulfamethoxazole against T. Pyogenes

**Figure S2** Concentration-time curves of sulfamethoxazole dosed at 2 and 4 mg/ml


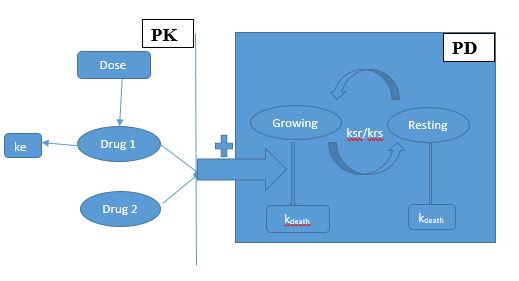


**Figure S3** Schematics of the semi-mechanistic (PK/PD) model characterizing the killing effect of aditoprim and sulfamethoxazole


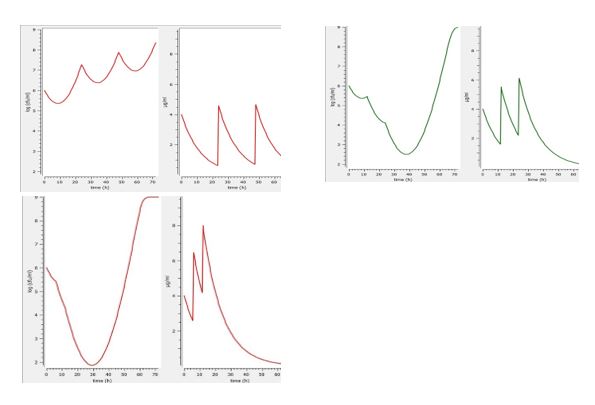


**Figure S4** Simulate different dosage regimens (2 mg/ml every 24 hr, 2 mg/ml every 12 hr and 4 mg/ml every 24 hr). The different doses were simulated for different intervals of time to find the efficient dose and dose intervals
